# Insulin-like growth factor binding protein-2: a new circulating indicator of pulmonary arterial hypertension severity and survival

**DOI:** 10.1186/s12916-020-01734-3

**Published:** 2020-10-06

**Authors:** Jun Yang, Megan Griffiths, Melanie K. Nies, Stephanie Brandal, Rachel Damico, Dhananjay Vaidya, Xueting Tao, Catherine E. Simpson, Todd M. Kolb, Stephen C. Mathai, Michael W. Pauciulo, William C. Nichols, David D. Ivy, Eric D. Austin, Paul M. Hassoun, Allen D. Everett

**Affiliations:** 1 Division of Pediatric Cardiology, Department of Pediatrics, Johns Hopkins University, 720 Rutland Ave. Ross RM 1143, Baltimore, MD 21205 USA; 2 Division of Pulmonary and Critical Care Medicine, Department of Medicine, Johns Hopkins University, Baltimore, MD USA; 3 Department of Epidemiology, Johns Hopkins Bloomberg School of Public Health, Baltimore, MD USA; 4 Division of General Internal Medicine, Johns Hopkins School of Medicine, Baltimore, MD USA; 5 Depart of Pediatrics, Biostatics Epidemiology and Data Management Core, Johns Hopkins School of Medicine, Baltimore, MD USA; 6 Division of Human Genetics, Cincinnati Children’s Hospital Medical Center; Department of Pediatrics, University of Cincinnati College of Medicine, Cincinnati, OH USA; 7 Department of Pediatric Cardiology, Children’s Hospital Colorado, Denver, CO USA; 8 Division of Allergy, Immunology, and Pulmonary Medicine, Department of Pediatrics, Vanderbilt University Medical Center, Nashville, TN USA

**Keywords:** Pulmonary arterial hypertension, Insulin-like growth factor binding protein 2, Biomarker, Survival

## Abstract

**Background:**

Pulmonary arterial hypertension (PAH) is a fatal disease that results from cardio-pulmonary dysfunction with the pathology largely unknown. Insulin-like growth factor binding protein 2 (IGFBP2) is an important member of the insulin-like growth factor family, with evidence suggesting elevation in PAH patients. We investigated the diagnostic and prognostic value of serum IGFBP2 in PAH to determine if it could discriminate PAH from healthy controls and if it was associated with disease severity and survival.

**Methods:**

Serum IGFBP2 levels, as well as IGF1/2 levels, were measured in two independent PAH cohorts, the Johns Hopkins Pulmonary Hypertension program (JHPH, *N* = 127), NHLBI PAHBiobank (PAHB, *N* = 203), and a healthy control cohort (*N* = 128). The protein levels in lung tissues were determined by western blot. The IGFBP2 mRNA expression levels in pulmonary artery smooth muscle cells (PASMC) and endothelial cells (PAEC) were assessed by RNA-seq, secreted protein levels by ELISA. Association of biomarkers with clinical variables was evaluated using adjusted linear or logistic regression and Kaplan-Meier analysis.

**Results:**

In both PAH cohorts, serum IGFBP2 levels were significantly elevated (*p* < 0.0001) compared to controls and discriminated PAH from controls with an AUC of 0.76 (*p* < 0.0001). A higher IGFBP2 level was associated with a shorter 6-min walk distance (6MWD) in both cohorts after adjustment for age and sex (coefficient − 50.235 and − 57.336 respectively). Cox multivariable analysis demonstrated that higher serum IGFBP2 was a significant independent predictor of mortality in PAHB cohort only (HR, 3.92; 95% CI, 1.37–11.21). IGF1 levels were significantly increased only in the PAHB cohort; however, neither IGF1 nor IGF2 had equivalent levels of associations with clinical variables compared with IGFBP2. Western blotting shown that IGFBP2 protein was significantly increased in the PAH vs control lung tissues. Finally, IGFBP2 mRNA expression and secreted protein levels were significantly higher in PASMC than in PAEC.

**Conclusions:**

IGFBP2 protein expression was increased in the PAH lung, and secreted by PASMC. Elevated circulating IGFBP2 was associated with PAH severity and mortality and is a potentially valuable prognostic marker in PAH.

## Background

Pulmonary arterial hypertension (PAH) is a fatal disease, characterized by increased pulmonary vascular remodeling and elevated mean pulmonary artery pressures [1, 2]. PAH is an extremely heterogeneous disease, classified into five clinical groups by the Sixth World Symposium on Pulmonary Hypertension (WSPH) in 2019 [3]. Although its precise etiology is not well understood, development of progressive pulmonary vascular resistance and associated right heart failure are common to all PAH patients [4, 5]. The treatment options for PAH have greatly expanded in the last half century, improving patients’ functional capacity and hemodynamics. However, PAH therapies focus on dynamic pulmonary vasoconstriction as the only mechanism of disease; thus, the 5-year mortality rate remains over 40% [6–8].

PAH is an insidious disease, with non-specific early symptoms, and which requires diagnosis by cardiac catheterization. Measurement of the commonly used blood marker N-terminal pro-brain natriuretic peptide (NTproBNP), a marker of cardiac dysfunction and increased stretch, is confounded by other factors such as left heart disease and renal function [9, 10]. What is urgently needed is a more pulmonary vascular specific, precise, and causally related biomarker which could improve non-invasive diagnosis, increase understanding of pathobiology, and improve non-invasive monitoring of disease progression.

The IGF axis consists of two hormones (IGF1 and 2), that bind with two types of receptors (IGFR1 and 2), and 6 binding proteins (IGFBP1-6) with high binding affinity to IGFs [11, 12]. Circulating IGFs usually form complexes with binding proteins, providing an IGF reservoir safe from degradation, but IGFBPs also directly affect cell function via IGF-independent mechanisms [13–18]. IGFBP2 in particular may play a specific role in lung function as circulating IGFBP2 levels were highly correlated with pulmonary fibrosis disease progression and treatment [19].

In a recent PAH plasma proteomics study, IGFBP2 was significantly increased compared to healthy controls [20]. In the current study, we measured IGFBP2 and total IGF1/2 levels in two independent PAH cohorts, as well as a healthy control cohort to evaluate the value of these proteins as diagnostic biomarkers for PAH; we then evaluated the relationships of these protein biomarkers with PAH progression and severity; finally, we evaluated IGFBP2 in the human PAH lung and pulmonary vascular cells.

## Methods

### Study cohorts

#### PAH patient cohorts

*The Johns Hopkins Pulmonary Hypertension (JHPH)* cohort is an independent cohort of adult patients that was enrolled through the JHPH Program (*n* = 127) (Table 1) [21]. Briefly, patients 18 years of age or older who were diagnosed with PAH by right heart catheterization were included. PAH subtypes were classified on the basis of guidelines defined by Sixth World Symposium on Pulmonary Hypertension. The diagnosis of connective tissue disease (CTD) was based on meeting either the American College of Rheumatology criteria, the presence of at least three of five features of the CREST syndrome (calcinosis, Raynaud’s phenomenon, esophageal dysmotility, sclerodactyly, and telangiectasia), or definite Raynaud’s phenomenon and the presence of a specific systemic sclerosis-related autoantibody [22]. The JHPH has maintained a registry since 2005 and sample collection started in 2007. Patients were followed prospectively until the data was censored for analysis in 2018. No patients were lost to follow-up. Survival was determined by review of the electronic record and search of the Social Security Death Index. Median follow-up was 4.2 years with a range of 0.2 to 10 years.

**Table 1:** Demographics and characteristics of all study cohorts

	JHPH cohort		PAHB cohort		Control cohort
*n*	127		203		128
Age (years)	62 (50–69)		56 (44–67)		43 (33–57)
Female (*n*, %)	107 (84%)		158 (78%)		96 (75%)
Race—EA, AA, other *n* (%)	95/20/12 (75%/16%/9%)		168/26/8 (83%/13%/4%)		105/15/8 (82%/12%/8%)
Co-morbidities (asthma /HTN/diabetes/thyroid)					6/20/3/6
IPAH/APAH/other, *n* (%)	46/81/0 (36%/64%/0%)		85/98/20 (42%/48%/10%)		
APAH-CTD	81 (64%)		71 (35%)		
APAH-PoPH			11 (5%)		
APAH-CHD			3 (1.5%)		
6MWD (m)	376	297–454	302	183–401	
NYHA-FC					
	*n* = 127	%	*n* = 144	%	
	I (13)	10%	I (7)	4%	
	II (53)	42%	II (49)	24%	
	III (60)	47%	III (75)	37%	
	IV (0)	0%	IV (13)	6%	
	No data (0)	0%	No data (59)	29%	
**Laboratory chemistries**					
NT-proBNP (pg/ml)	779.5	(249.5–2826.6)	1253.5	(369.9–3593.3)	
**Hemodynamics**					
RAP (mmHg)	7 (4–9)		9 (6–14)		
mPAP (mmHg)	39 (29–50)		48 (40–56)		
PCWP (mmHg)	10 (7–12)		11 (8–13)		
PVR (WU)	6.4 (3.4–10.3)		9.4 (6.8–13.9)		
CO (L^.^min^−1^)	4.4 (3.7–5.5)		4 (3.2–4.9)		
CI (L^.^min^−1^/m^−2^)	2.5 (2.1–3.1)		2.2 (1.7–2.8)		

Data expressed as median and IQR, number (*n*), percentage (%), or range as indicated

*Abbreviations*: *EA* European American, *AA* African American, *HTN* hypertension, *thyroid* thyroid disease, *RAP* right atrial pressure, *mPAP* mean pulmonary artery pressure, *PCWP* pulmonary capillary wedge pressure, *PVR* pulmonary vascular resistance, *WU* Wood units, *CO* cardiac output, *CI* cardiac index, *NYHA-FC* New York Heart Associated-Functional Class, *6MWD* 6-min walk distance, *NT-proBNP* N-terminal pro-brain natriuretic peptide, *JHPH* Johns Hopkins Pulmonary Hypertension, *PAHB* Pulmonary Arterial Hypertension Biobank

*National Biological Sample and Data Repository for Pulmonary Arterial Hypertension, or PAH Biobank (PAHB)* is a NHLBI-funded resource of WSPH Group 1 PAH patient biological samples, genetic data, and clinical data enrolled from 38 US Centers (www.pahbiobank.org). Under an approved protocol, we analyzed all PAHB enrollees with a cardiac catheterization within 6 months of enrollment (*N* = 203) for contemporaneous comparison of biomarkers to hemodynamics. The median follow-up was 40.5 months (IQR 22.7–49.7). After enrollment, 36 enrollees were lost to follow-up. Their demographic and clinical information are summarized in Table 1.

Patients with interstitial pulmonary fibrosis were excluded from both cohorts.

#### Control cohort

Serum from healthy adult volunteers was collected at three independent research centers: Johns Hopkins Pulmonary Center, Johns Hopkins Anesthesia Safety Study [23], and Vanderbilt University Medical Center. A total of 128 volunteers participated, with demographic information summarized in Table 1.

### IGF1, IGF2, and IGFBP2 measurements in serum

Total IGF1, IGF2, and IGFBP2 serum levels were measured using commercial ELISA kits (R&D, Cat # DG100, Human IGF-I Quantikine ELISA Kit; Cat # DG200, Human IGF-II Quantikine ELISA Kit; Cat # DGB200, Human IGFBP-2 Quantikine ELISA Kit). Total IGF1 and 2 measurements (free and bound) were determined by pre-treatment of the serum samples as per manufacturer protocol. All assays required the serum samples to be properly diluted (dilution factors were 100, 2000, and 50 respectively). All ELISA procedures and data analysis were performed in the same lab and followed the manufacturer protocols.

### Lung tissue, Western blotting

Explanted lung tissues were provided by University of Alabama, under the Pulmonary Hypertension Breakthrough Initiative (PHBI), funded by the Cardiovascular Medical Research and Education Fund (CMREF). Lung tissues used in this study were from 4 donor lung explants and 4 IPAH patients. Proteins were extracted from lung tissues by homogenization of the tissues in protein extraction buffer (8 M urea, 2 M thiourea, 4% CHAPS, 1% DTT). Proteins were cleared by centrifugation and the total protein was quantified using the Pierce 660 nm protein assay reagent (Cat# 22660, ThermoFisher). The Western blotting protocol was described previously [24]; in brief, rabbit polyclonal antibody specified for human IGFBP2 (Cat# 3922, Cell Signaling, MA) was used to detect IGFBP2 protein. A mouse monoclonal antibody for actinin (Cat# MAB1682, Sigma) was used as loading control.

### Primary cell culture conditions

Primary small pulmonary artery smooth muscle (PASMC) and endothelial cells (PAEC) were obtained from PHBI cell core facility (University of Pennsylvania) funded by CMREF. The cells were isolated from small pulmonary arteries of transplanted patients with severe PAH or donor lungs. All cells were maintained at low passage (passage 3–8) and in cell-specific culture media for PASMC (VascuLife SMC Medium, Cat # LL-0014, Lifeline Cell Technology, Frederick, MD) and PAEC (VascuLife VEGF-Mv Endothelial Medium, Cat # LL-0005, Lifeline Cell Technology, Frederick, MD). After 24 h incubation, conditioned media was harvested and centrifuged at 3000 rpm for 5 min at 4 °C; the supernatants were aliquoted and stored at − 80 °C, until assayed for IGFBP2 by ELISA. The IGFBP2 media concentration was normalized to the cell total protein concentration.

### RNA extraction and RNAseq analysis

We used TRIzol reagent (Cat# 15596026, ThermoFisher Scientific) to extract total RNA from PAECs and PASMCs in standard culture condition at 80–90% confluency for RNAseq experiments. RNAseq library preparation, quantification, and sequencing was performed commercially (ArrayStar 6G RNAseq service, Rockville, MD). In brief, the libraries were sequenced for 150 cycles for both ends (Illumina NovaSeq 6000). Image analysis and base calling were performed using Solexa pipeline v1.8 (Off-Line Base Caller software, v1.8). Sequence quality was examined using the FastQC [25] software. Hisat2 software [26] was used to align the trimmed reads to reference genomes. The transcript abundances for each sample was estimated with StringTie [27], and the FPKM [28] value for gene and transcript level were calculated with the R package Ballgown [29–31]. IGFBP2 gene expression levels (FPKM values) were extracted from the data analysis results.

### Statistical analysis

IGF1, IGF2, and IGFBP2 levels and demographic and functional test data are presented as median and interquartile range (IQR), or number and percentage, where appropriate. Because the values were skewed, we studied the association of logarithmically transformed serum IGFBP2 and IGFs with various clinical measures using unadjusted tests, spearman’s rank correlation test for continuous variables and Kruskal-Wallis test for categorical variables, and used age- and sex-adjusted regression analysis, linear for continuous variables or logistic for dichotomous variables. Youden analysis was used to determine the best threshold value to distinguish the PAH from healthy controls based on maximum of sum of sensitivities and specificities as the optimality criterion. We examined the association of IGFBP2 and IGF levels dichotomized at the median for survival using unadjusted Kaplan-Meier analysis and Cox proportional hazard regression analysis adjusted for age, sex, NYHA-FC, hemodynamics (right atrial pressure [RAP], pulmonary artery pressure [PAP], pulmonary vascular resistance [PVR]), PAH type, 6-min walk distance (6MWD), and body mass index (BMI). A *p* value less than 0.05 was considered statistically significant. Statistical analysis was performed using STATA (Version 15, StataCorp LLC, College Station, TX) and MedCalc (2019 version; MedCalc Software, Ostend, Belgium).

## Results

### IGF1, IGF2, and IGFBP2 in PAH

#### JHPH cohort

The demographic information and clinical characteristics of the JHPH cohort (*N* = 127) are summarized in Table 1. The serum samples of all patients were obtained at a single time point (enrollment). The median age was 62 years old, and 84% were women. Thirty-six percent of the patients were diagnosed with idiopathic pulmonary hypertension (IPAH) and 64% with PAH associated with connective tissue disease (APAH-CTD). The overall mortality was 43%, with 54 deaths during the follow-up period of 5 years.

**Fig. 1:**
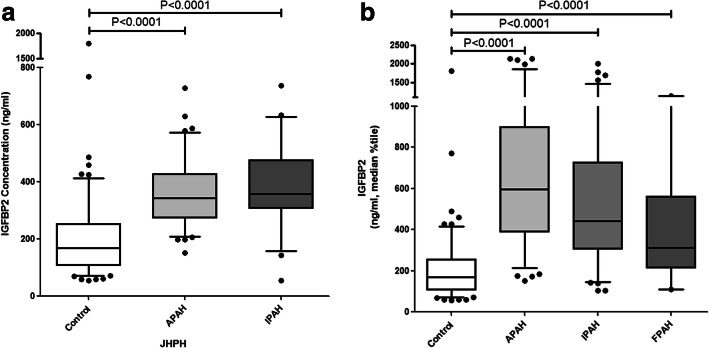
Box and whisker plot of serum IGFBP2 levels in subgroups of APAH (*N* = 81) and IPAH (*N* = 46) in JHPH cohort (**a**) versus healthy control (*N* = 128). Boxes represent the interquartile range (IQR 5–95%) and horizontal lines are the median. Outliers are indicated with solid dots. **b** The subgroups of APAH (*N* = 98), IPAH (*N* = 85), and FAPH (*N* = 20) in the PAHB cohort in the same style

As shown in Table 2, the circulating IGFBP2 concentration was significantly increased in PAH in aggregate compared with healthy control subjects (median 350.9 ng/ml vs 170.2 ng/ml, *p* < 0.0001) or in the PAH subtypes IPAH (median 358.4 ng/ml, *p* < 0.0001) and APAH (median 344.2 ng/ml, *p* < 0.0001) versus healthy control (Fig. 1a), although no significant differences were observed for IGF1 (median 67.0 vs 64.7 ng/ml, *p* = 0.15) or IGF2 (347.8 vs 340.9 ng/ml, *p* = 0.56) versus PAH in aggregate.

**Table 2:** Circulating IGF axis proteins levels

	JHPH, ***N*** = 127	PAHB, ***N*** = 203	Control, ***N*** = 128
	mean ± SD, ***p*** value	mean ± SD, ***p*** value	mean ± SD
	(median; range)	(median; range)	(median; range)
**IGF1 (ng/ml)**	72.9 ± 30.4, *p* = 0.15	89.6 ± 51.3, *p* < 0.0001	69.4 ± 34.4
	(67.0; 24.3–222.8)	(79.1; 21.6–486.6)	(64.7; 18.3–199.6)
**IGF2 (ng/ml)**	374.5 ± 121.9, *p* = 0.56	400.3 ± 182.8, *p* = 0.18	385.0 ± 171.6
	(347.8; 56.1–908.1)	(391.6; 55.4–963.4)	(340.9; 56.6–994.5)
**IGFBP2 (ng/ml)**	419.8 ± 307.4, *p* < 0.0001	621.2 ± 425.9, *p* < 0.0001	207.3 ± 180.5
	(350.9; 55.5–1869)	(474.6; 103.6–2144)	(170.2; 55.6–1806)

*p* value was calculated by Mann-Whitney test between the values from PAH and control cohorts

#### PAHB cohort

NHLBI PAHbiobank (PAHB, *N* = 203) is a multi-center larger cohort. The demographic data was similar to the data from the JHPH cohort and is summarized in Table 1. The median age was 56 years old, and 78% were female, 42% with IPAH and 48% with associated pulmonary arterial hypertension (APAH), 35% APAH-CTD. Overall mortality was 23%, with 47 deaths during the 5-year follow-up period and 36 lost to follow-up.

In the PAHB cohort, the circulating IGFBP2 concentration was significantly increased compared with healthy control subjects (median 474.6 vs 170.2 ng/ml, *p* < 0.0001). Compared to controls, the IGFBP2 concentration was also significantly increased in IPAH (median 441.1 ng/ml, *p* < 0.0001), APAH (median 594.9 ng/ml, *p* < 0.0001), and FPAH (median 312.5 ng/ml, *p* < 0.0001) subgroups (Fig. 1b). IGF1 was also significantly increased (median 79.1 vs 64.7 ng/ml, *p* < 0.0001), but no significant difference existed for IGF2 (391.6 vs 340.9 ng/ml, *p* = 0.18) (Table 2).

### IGFBP2 discriminates PAH from healthy controls

**Fig. 2:**
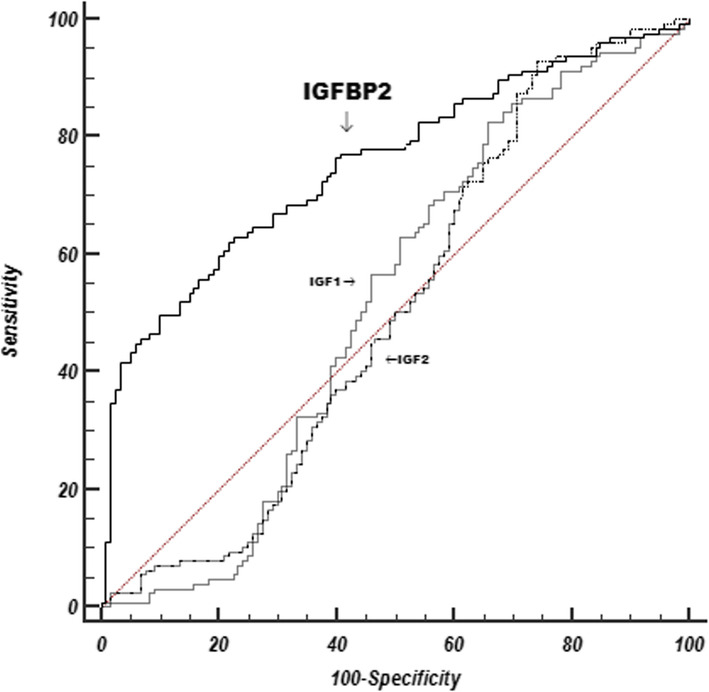
Comparison of receiver operating curves (ROC) for IGF1, IGF2, and IGFBP2as predictors of pulmonary arterial hypertension for 127 subjects from the JHPH cohort and 128 healthy control subjects. The AUC for IGFBP2 is 0.76, 95% confidence interval [CI] 0.698–0.808, *p* < 0.0001; AUC for IGF1 is 0.52, 95% confidence interval [CI] 0.455–0.583, *p* = 0.61; and AUC for IGF2 is 0.50, 95% confidence interval [CI] 0.438–0.566, *p* = 0.96

In order to evaluate whether IGF axis proteins are able to discriminate patients with PAH from healthy controls, we generated ROC curves for IGF1, IGF2, and IGFBP2, using their values from JHPH and control cohorts. As shown in Fig. 2, among the three ROC curves, IGFBP2 was the best performer with an AUC of 0.76 (95% confidence interval [CI] 0.698–0.808, *P* < 0.0001). A serum IGFBP2 cutoff value was established by Youden analysis at 262.8 ng/ml to distinguish PAH from controls. This cutoff value had a sensitivity and specificity for PAH of 62.2% and 78.5% respectively. The performance of this cutoff value was then tested with the PAHB cohort and healthy controls. In this analysis with a case prevalence of 62%, the test performed well in discriminating PAH, with positive and negative predictive values of 84% and 79% respectively. For IGFBP2 values less than 262.8 ng/ml, the likelihood ratio for the presence of PAH was 0.2 (95% confidence interval [CI] 0.15–0.28), while for values of 262.8 or greater, the likelihood ratio was 3.9 (95% CI 2.79–5.58).

### IGFBP2 correlates with PAH severity

We determined the relationship between serum IGF1, IGF2, and IGFBP2 and invasive resting hemodynamics, exercise tolerance assessed by 6MWD, and functional class. Significant negative correlation of IGFBP2 with 6MWD was observed in both JHPH (*r* = − 0.223, *p* = 0.018) and PAHB (*r* = − 0.314, *p* < 0.001) cohorts (Table 3). Using linear regression analysis adjusted for age and sex, each log-unit increase of IGFBP2 was associated with a 50 and 57 m decrease in 6MWD in JHPH (coefficient − 50.235, *p* = 0.16) and PAHB (coefficient − 57.336, *p* = 0.012) respectively. Each log-unit increase in IGFBP2 was associated with a 9-mmHg higher mean pulmonary arterial pressure (PAP) in the JHPH cohort (coefficient 9.103, *p* = 0.026). In the PAHB cohort, each log-unit increase in IGFBP2 was associated with a 0.89-mmHg lower pulmonary capillary wedge pressure (PCWP) (coefficient − 0.89, *p* = 0.023, Table 4). When we studied subtypes of PAH, IGFBP2 was significantly associated with APAH-CTD in both JHPH and PAHB cohorts (rank sum *p* < 0.0001 for both cohorts); using logistic regression analysis after adjusting for age and sex, the significant association was only found in the PAHB cohort (coefficient 0.958, *p* = 0.001) (Tables 3 and 4).

**Table 3:** Associations (unadjusted) between IGFBP2 and clinical variables

	**JHPH**		**PAHB**	
*Spearman Rank Correlation*	*Spearman Rho*	*p-value*	*Spearman Rho*	*p-value*
**Age**	***0.527***	***<0.0001***	***0.384***	***<0.0001***
**BSA (Body Surfae Area)**	***-0.233***	***0.013***	***-0.153***	***0.034***
**SixMinWalkDist (meters)**	***-0.223***	***0.018***	***-0.314***	***0.001***
**Cardiac output**	-0.1	0.265	-0.111	0.116
**Cardiac index**	-0.033	0.769	-0.043	0.552
**PVR**	0.123	0.171	0.062	0.387
**mPAP**	0.112	0.212	-0.036	0.609
**mRAP**	0.036	0.685	0.124	0.082
**mPCWP**	0.035	0.696	-0.113	0.111
*Kruskal Wallis Test*	*p-Value*		*p-Value*	
**NYHA-FC**	0.13		0.307	
**REVEAL**			***<0.0001***	
**APAH-CTD**	***<0.0001***		***<0.0001***

**Table 4:** Associations (adjusted for age and sex) between IGFBP2 and clinical variables

	JHPH		PAHB	
	Coefficient	***p*** value	Coefficient	***p*** value
**Linear regression**				
**BSA (body surface area)**	− 0.144	0.058	***− 0.072***	***0.014***
**SixMinWalkDist (meters)**	**− 50.235**	**0.16**	***− 57.336***	***0.012***
**Cardiac output**	− 0.292	0.554	− 0.224	0.236
**Cardiac index**	1.284	0.605	− 0.039	0.722
**PVR**	2.811	0.07	1.175	0.062
**mPAP**	***9.103***	***0.026***	0.913	0.529
**mRAP**	1.251	0.33	1.289	0.056
**mPCWP**	0.207	0.835	***− 0.89***	***0.023***
**Logistic regression**				
**NYHA-FC**	0.783	0.22	0.436	0.135
**APAH-CTD**	1.166	0.099	***0.958***	***0.001***

The relationships between IGF1/2 and the clinical variables were either not significant or not consistent between the two cohorts. There was also no significant association between any of the IGF axis proteins with functional class (Tables 3 and 4, Supplemental Table 1–4).

### IGFBP2 and survival in PAH

The REVEAL score, a validated predictive algorithm for 1-year survival in PAH, was used to calculate mortality risk [32, 33]. In the PAHB cohort, IGF2 and IGFBP2 were both significantly associated with an increasing REVEAL score (rank sum *p* = 0.028 and *p* < 0.0001 respectively, Supplemental Table 2, Table 3).

To evaluate the survival predictive power, the Harrell’s c index of the REVEAL score and IGFBP2 plus REVEAL score were calculated. Compared to the model using REVEAL score to predict survival (Harrell’s c index = 0.626), the model using both REVEAL score and IGFBP2 (Harrell’s c index = 0.716, *p* < 0.001) had better predictive power.

**Fig. 3:**
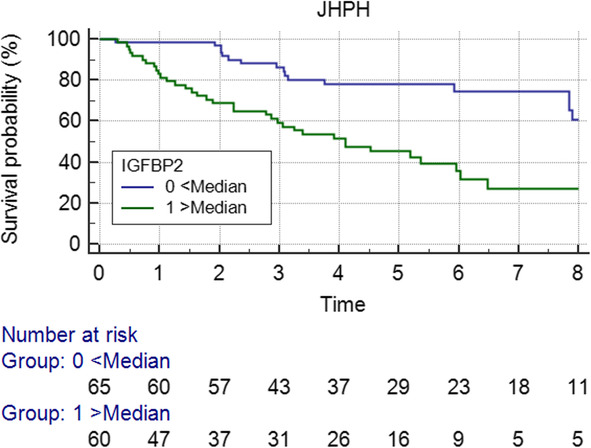
Kaplan-Meier survival curve for IGFBP2 in the JHPH cohort. The curves represent survival analysis of JHPH cohort dichotomized by median serum IGFBP2 (*n* = 125, *p* = 0.0001) concentration

We assessed the relationship between IGF axis protein levels and mortality using Kaplan-Meier analysis in JHPH and PABH cohorts. In the JHPH cohort, elevated IGFBP2 levels above the median value were significantly associated with an increased risk of death, with an unadjusted hazard ratio of 2.97 (95% CI 1.77–5.3; *p* = 0.0001 by log-rank test) (Fig. 3). However, neither IGF1 nor IGF2 was significantly associated with mortality in the JHPH cohort (*p* = 0.82 for IGF1; *p* = 0.23 for IGF2).

**Fig. 4:**
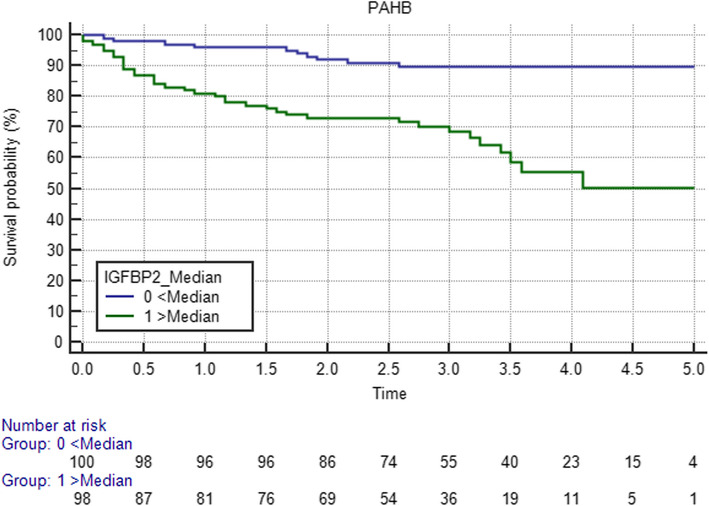
Kaplan-Meier survival curve for IGFBP2 in the PAHB cohort. The curves represent survival analysis of PAH cohort dichotomized by median serum IGFBP2 (*n* = 200, *p* < 0.0001) concentration

In the PAHB cohort, similar results were observed with IGFBP2 levels above the median value significantly associated with an increased risk of death, with an unadjusted hazard ratio of 3.95 (95% CI, 2.2–7.1; *p* < 0.0001 by log-rank test) (Fig. 4). Decreased concentrations of both IGF1 and IGF2 were also significantly associated with risk of death (HR 2.5, 95% CI, 1.41–4.42; *p* = 0.002 for IGF1; HR 2.9, 95% CI, 1.64–5.13; *p* = 0.0002 for IGF2).

We constructed a Cox multivariable proportional hazard model, adjusted for significant clinical variables: age, sex, NYHA-FC, hemodynamics (RAP, PAP, PVR), PAH type, 6MWD, and BMI to examine the relationship between IGF axis proteins and survival. In the PAHB cohort, increased serum IGFBP2 predicted decreased survival, with a hazard ratio [HR] of 3.92 (95% CI, 1.37–11.21; *p* = 0.011), but not significant in the JHPH cohort, HR 2.64 (95% CI, 0.7–10.04; *p* = 0.15).

In PAHB cohort, there were 36 patients lost follow-up, although their data were censored, their survival status was unknown. To test whether these incomplete data affected the association of IGFBP2 and mortality, we did sensitivity analysis, by removing these 36 patients’ data completely. COX analysis shown that HR decreased to 3.21 (95% CI 1.09–9.39, *p* = 0.033), indicating that the association between IGFBP2 and mortality was not affected.

### IGFBP2 protein highly expressed in the lung tissues of PAH patients

**Fig. 5:**
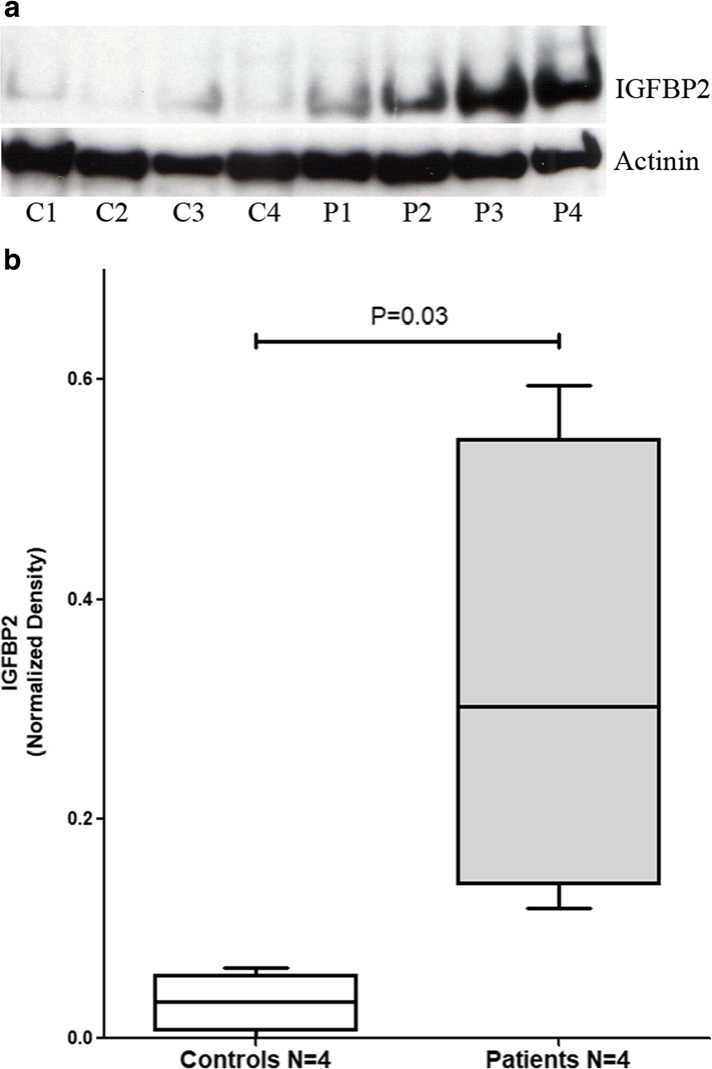
IGFBP2 protein levels in the lung tissues measured by western blot using IGFBP2 specific antibody or actinin level as a loading control (**a**). C1–C4 are control donor lung samples, and P1–P4 are lung samples from PAH patients. Box and whisker plot of IGFBP2 protein levels in lung samples from PAH patients versus from controls (**b**). Boxes represent the interquartile range (IQR 5–95%) and horizontal lines are the median. The IGFBP2 protein level in each sample (bands in **a**) was quantified using ImageJ software

To evaluate the protein expression level of IGFBP2 in the PAH lung, we performed Western blot analysis on lung tissue protein extracts from 4 donors and 4 IPAH patients. As shown in Fig. 5, IGFBP2 was detected as a specific ~ 35 Kd band with IGFBP2 protein detected in all the lung tissue samples. IGFBP2 protein levels were significantly higher in PAH patients than that in the donor lungs, after normalization for protein loading (Fig. 5b).

### Both PAEC and PASMC secret IGFBP2

**Fig. 6:**
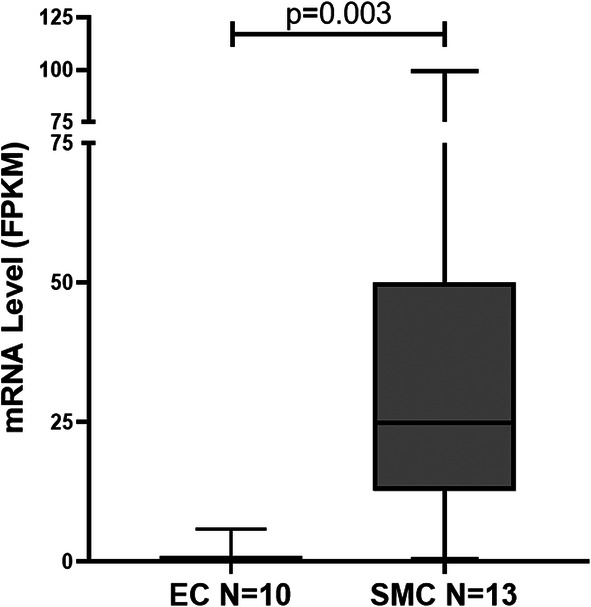
Box and whisker plot of IGFBP2 mRNA levels in PAEC (*N* = 10) versus that in PASMC (*N* = 13). Boxes represent the interquartile range (IQR 5–95%) and horizontal lines are the median

To test whether the pulmonary arterial cells are a source of IGFBP2 in the lung, we tested the mRNA expression levels of IGFBP2 in cultured PAEC and PASMC, using RNA-seq. A total of 10 PAEC and 13 PASMC separate cell lines were analyzed. As shown in Fig. 6, the IGFBP2 mRNA levels in PASMC were significantly higher than in PAEC but not significantly different between PAH and controls.

**Fig. 7:**
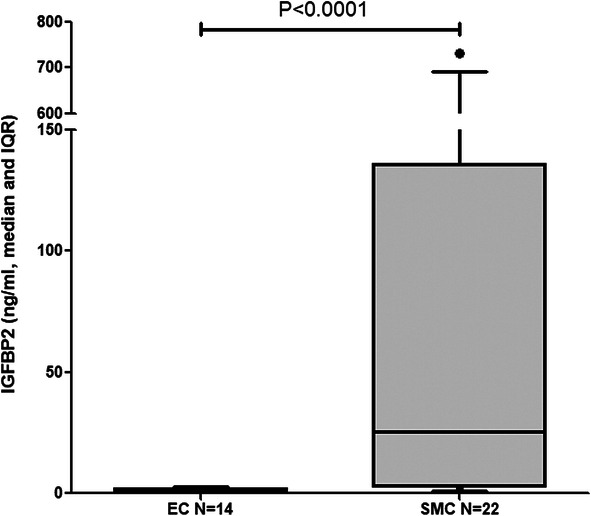
Box and whisker plot of IGFBP2 protein levels in conditional medias from PAEC (*N* = 14) versus that in PASMC (*N* = 22); IGFBP2 level was normalized by cultured cell protein concentration. Boxes represent the interquartile range (IQR 5–95%) and horizontal lines are the median

We further measured IGFBP2 protein levels in the condition media from both PAEC and PASMC using the same ELISA method as used for serum analysis. IGFBP2 was secreted from both PAEC and PASMC, but the level was significantly higher in PASMC (Fig. 7).

## Discussion

In a recent proteomic discovery study, IGFBP2 was identified to be elevated in PAH [20]. We tested and verified that IGFBP2 was markedly increased in PAH using 2 independent PAH cohorts, a Johns Hopkins PAH single center cohort and a multicenter PAH cohort (NHLBI PAHBiobank). Using these cohorts, we demonstrated that IGFBP2 was significantly associated with PAH, survival, and disease severity (REVEAL score, 6MWD). We also demonstrated that IGFBP2 is increased in the PAH lung, expressed and actively secreted by PASMC.

IGFBP2 is a member of a large family of six binding proteins for IGF1 and IGF2 [11, 12]. In general, circulating IGF1 and 2 are bound to IGFBPs, as free IGFs are rapidly degraded. IGFBPs prevent IGF degradation and facilitate delivery of IGFs to the IGF cell surface receptors to trigger an essential IGF growth signal [11, 12]; however, some IGFBPs, including IGFBP2, have been shown to stimulate cell growth in an IGF-independent manner [16, 18].

Circulating IGFBPs have also been associated with other cardiopulmonary diseases [19, 34–36]. For example, an aptamer panel screening study found that IGFBP1 was one of several elevated proteins in non survivors of adult PAH patients, which improved the predictability of mortality by REVEAL risk score [34]. In adults with pulmonary fibrosis, another disease process that often complicates the collagen vascular diseases associated with PAH, IGFBP2 was found to be increased, more interestingly, serum IGFBP2 level was reduced by antifibrotic and/or anti-inflammatory therapy, and inflammation is an important component of PAH pathology [19, 37].

IGFBP2 is the second most abundant IGF binding protein. Targeted IGFBP2 knock-down in zebrafish embryos demonstrated angiogenic defects, in particular, cardiovascular development disruption, reduced blood cell number, cardiac dysfunction, and brain ventricle edema [38]. IGFBP2 plays an important role in the IGF growth axis, with elevated levels associated with lower fasting insulin and fasting glucose, but with greater mortality in older adults and in patients with dilated cardiomyopathy [39, 40]. In the present study, we found IGFBP2 serum levels to be significantly increased in PAH patients and associated with increased risk for death. We also found that IGFBP2 was increased in the IPAH lung and is highly expressed and released by PASMC. Although the function of IGFBP2 in angiogenesis and cardiac development remains elusive, this study suggests that IGFBP2 contributes to vascular function in PAH; IGFBP2 may be a key factor for PAH development and may give insight into new treatments.

IGFBP2 is overexpressed in many tumors, especially in blood vessel growth into large tumors, and IGFBP2 expression levels are highly correlated with grade of malignancy and poor tumor differentiation [41–43]. Recent studies have found that IGFBP2 is an essential component for maintaining ex vivo expansion of hematopoietic stem cells (HSC) [44, 45]. In vascular smooth muscle cells, Clemmons group demonstrated that IGFBP2-potentiated IGF1 induced VSMC migration and proliferation through interaction with receptor protein tyrosine phosphatase β (RPTPβ) which led to RPTPβ dimerization and inactivation, which in turn cause PTEN (phosphatase and tensin homolog deleted on chromosome 10) phosphorylation and loss of function [46]. Importantly, a genetic mouse model with conditional knockout of PTEN in smooth muscle cells demonstrated spontaneous development of pulmonary hypertension, with increasing Akt activity in major vessels, heart, and lungs, and widespread medial SMC hyperplasia with vascular remodeling [47]. In our study, we found IGFBP2 was increased significantly in all PAH cohorts. It is intriguing to hypothesize that increased circulating IGFBP2 may contribute to PAH pathogenesis through downregulation of PTEN and its underlying regulatory mechanism.

There are some limitations for this study. First, the PAHB cohort was from a multi-center registry, with some covariates missing or incomplete. For example, 36 patients had incomplete mortality data. However, sensitivity analysis demonstrated a minimal impact on the overall result. Serum collection time usually was not contemporaneous with the assessments of other clinical variables; to minimize this effect, we only included subjects with a cardiac catheterization assessment performed within 6 months of enrollment. PAH is an extreme heterogeneous disease; different subtypes may have very different pathology. Ultimately, the performance of IGFBP2 as a biomarker will need to be studied in larger cohorts.

## Conclusions

To summarize this study, IGFBP2 is a potential new circulating PAH biomarker associated with disease severity and survival and provides valuable clinical prognostic information. The increased expression of IGFBP2 in PAH lung and PASMC suggests that IGFBP2 could have a direct role in pulmonary pathobiology of PAH. Further improved understanding of this new pathway is needed to support the possible utility development for PAH clinical care.

## Supplementary information

**Additional file 1: Supplemental Table 1.** Associations (Unadjusted) between IGF1 and clinical variables. **Supplemental Table 2.** Associations (Unadjusted) between IGF2 and clinical variables.

**Additional file 2: Supplemental Table 3.** Associations (adjusted for age and sex) between IGF1 and clinical variables. **Supplemental Table 4.** Associations (adjusted for age and sex) between IGF2 and clinical variables.

## Data Availability

Dataset available on request to the corresponding authors (Dr. Everett, Dr. Yang)

## Abbreviations

IGFBP2: Insulin-like growth factor binding protein-2
IGF1/2: Insulin-like growth factor 1/2
PAH: Pulmonary arterial hypertension
IPAH: Idiopathic pulmonary hypertension
APAH: Associated pulmonary arterial hypertension
CTD: Connective tissue disease
CHD: Congenital heart disease
HTN: Hypertension
JHPH: Johns Hopkins Pulmonary Hypertension program
PAHB: NHLBI PAHBiobank
PASMC: Pulmonary artery smooth muscle cells
PAEC: Pulmonary artery endothelial cells
AUC: Area under the curve
6MWD: 6-min of walk distance
NYHA-FC: New York Heart Association functional classification
RAP: Right atrial pressure
PAP: Pulmonary artery pressure
PVR: Pulmonary vascular resistance
PHBI: Pulmonary Hypertension Breakthrough Initiative
CMREF: Cardiovascular Medical Research and Education Fund
NTproBNP: N-terminal pro-brain natriuretic peptide
ELISA: Enzyme-linked immunosorbent assay
FPKM: Fragments Per Kilobase of transcript per Million mapped reads
IPF: Idiopathic pulmonary fibrosis
REVEAL: Registry to Evaluate Early and Long-Term PAH Disease Management
HSC: Hematopoietic stem cells
PTEN: Phosphatase and tensin homolog deleted on chromosome 10
RPTPβ: Receptor protein tyrosine phosphatase β
BSA: Body surface area
BMI: Body mass index
